# Clinical Diagnosis of Tumors

**Published:** 1899-04

**Authors:** Robert Formad

**Affiliations:** Philadelphia, Pa.


### CLINICAL DIAGNOSIS OF TUMORS.

BY ROBERT FORMAD, V.M.D.,
PHILADELPHIA, PA.

THE study of tumors constitutes one of the most interesting as well as useful chapters in pathology. The interest is not only from the stand-point of teaching or the examination-room, but also from the practical application for the busy practitioner and surgeon, who is concerned not in the latest views, not in the charms of the microscopical picture that a section of the tumor shows to the pathologist, but he is more interested whether that tumor will

[1] Read at the annual meeting of the Pennsylvania State Veterinary Medical Association, March, 1899.

return or not return after removal, and whether that tumor has given or not given any metastasis to internal organs, and after the operation has been successfully performed the metastatic nodules continue to grow with renewed vigor and spoil the result of a successful operation. Your committee of arrangements in assigning me to give this demonstration impressed on me in the most forcible manner that this should be not a scientific lecture, for which you have no time, but a demonstration, and in presenting to you this subject I will endeavor to make it as practicable as possible. A few brief remarks, definitions, and classifications are, however, indispensable to treat the subject in a systematic manner. These remarks, I hope, will show that even the most scientific surgeon in performing an operation does not need to hold the scalpel in one hand and the microscope in the other hand, to have success; but every surgeon before plunging the knife into the tissues, even of the dumb beast, must bear in mind that there are benign and malignant tumors, and that the recidivity would greatly influence his prognosis, even if he cannot recall the exact microscopical picture. When we treat disease it is essential to observe and know not only the symptoms and peculiarity, but also the name of the disease to which the symptoms belong, and when we wish to remove a tumor we must remember the kinds of tumors that pathology teaches.

Tumors are studied under the heading of composite morbid processes, termed pseudo-formations, leading to the development of useless tissue in contrast to regeneration, the term new-tissue formation.

*Definition.* (*a*) Clinical: A tumor is a swelling developed without the symptom of inflammation; it is permanent, and has a tendency to stay and increase in size. (*b*) Anatomical: A tumor is a multiplication of pre-existing tissue above physiological limits, abnormal as to space and tissue.

*Synonyms.* Hyperplasia, swelling, neoformation, neoplasm.

Inflammatory new-formation may resemble tumors to such an extent as to make the distinction an exceedingly difficult one, even on a morphological and physiological basis. Tumors originate spontaneously, but do not disappear so, while inflammatory neoformations do not appear spontaneously, but may disappear so.

*Pathogenesis.* Numerous theories have been advanced from time to time to explain the causes of tumors, but up to the present day our theories have been hypotheses failing to explain the causation of tumors.

1. The dyscrasia or diathesis theory, advanced by Billroth and older writers, ascribed formation of tumors to a diseased state of the fluids of the body and an inherited peculiarity. If this be true, however, the question naturally would arise, When did the first tumor start? If Adam was created with the first tumor, it is strange that a tumory nation has not been developed.

2. The mechanical or inflammatory theory comes next, and ascribes the formation of tumors to irritants—*e. g.*, epithelioma in pipe-smokers, and to injuries. Clinical observation shows this to be true in a number of sarcomas and carcinomas; but not every injury leads to tumors, and if it would, not many of our forefathers would have reached a good old age, as in those days the mode of bringing up was quite different and more forcible than now; athletics would never have reached the present state of development; we would be deprived the pleasure of seeing the pleasant countenance of Dr. John Adams and Dr. Senseman if this mechanical theory was unimpeachable.

3. The embryonal or evolutional theory of Cohnheim ascribes the formation of tumors to errors of development during embryonal life, when certain cells having been displaced develop the tissue for which they were intended, even if these cells have been moved to another locality. It is the only way in which we can account for the formation of certain congenital tumors, as dermoid cysts or certain mixed tumors. It has more limited application than the preceding ones.

4. The nervous theory ascribes the formation of tumors to disturbances in the trophic functions of the nervous system, but is more of an imaginary hypothesis than a reality.

5. The parasitic theory assumes that parasites are responsible for the formation of tumors, but it will be hypothetic as the parasites are not demonstrated.

Classification of tumors:

I. By nature (whether benign or malignant).

II. By shape; this depends upon the manner of growth, situation, and the influence of the surrounding parts.

III. By structure or histogenetic classification, which is most convenient for a systematic study in the laboratory.

I. *Classification by nature:* Tumors are benign or malignant. The former (benign) do not affect the general health of the patient in any notable degree, and are dangerous mainly by reason of the pressure they exert on the vital structures, on the secondary causes (hemorrhage, suppuration, softening) to which they are liable. Malignant tumors disturb the general health from the first, tend to recur after removal, and spread to other parts by metastasis or direct invasion, secondary particles being carried through the circulation or the lymphatic channels.

Each of these two groups of tumors has its own peculiarity, which can be summed up as follows: Benign: Have a capsule; are homologous; usually poor in bloodvessels; no metastasis; generally multiple; may grow very large; ulcer, if exists only superficially, in the skin (lipoma); do not recur; never kill except mechanically; no cachexia. Malignant: Have no capsule; are heterologous; usually soft and juicy; rich in bloodvessels; give metastasis; generally single (primary, except spindle-cell sarcoma, which may be multiple); primary always small; secondary may grow large; often ulcerate; tumor itself being involved do recur; kill by destruction of tissues and metastasis; show cachexia, progressive emaciation from lack of nutrition. The malignant tumors are represented by the various forms of cancer and sarcomas; all the rest are benign, with the exception of the myxoma, which may be either.

II. *Classification by shape:*

1. Uniform swelling: Goitre, lymphoma, glioma.

2. Nodes growing centrally (all benign which grow below the surface): Fibroma, myoma, lipoma, chondroma, neuroma, etc.

3. Nodes by infiltration, sending roots: All malignant tumors, sarcoma, and cancer.

4. Desquamation: Ichthyosis.

5. Flat tubular swelling: Benign angioma, lymphoma, keloid; malignant epithelioma, squamous and cylindrical.

6. Hemispherical growths: Multiple fibroma and spindle-celled sarcoma.

7. Tuber: Chondroma, osteoma, osteochondroma.

8. Papilla: Horns, corns, chondyloma (pointed).

9. Fungus rich in color and not covered by skin: Telangiastatic sarcoma and carcinoma.

10. Polyp or pedunculated growth: Myoma, soft fibroma, adenoma.

11. Dendritic or cauliflower growth: Papilloma, if growing only upward; epithelioma, if growing both upward and inward.

12. Cysts: Hollow tumors.

III. *Histogenetic classification:*

*A.* Histioid tumors. *a.* Perfect: Fibroma, repeating the structure of fibrous tissue; myoma, mucous tissue; lipoma, adipose tissue; osteochondroma, callus tissue; osteoma, bone; myoma, muscle; neuroma, nerve; angioma, bloodvessels; lymphangioma, lymph-vessels. *b.* Imperfect: All the forms of sarcoma and glioma.

The perfect histoid tumors are formed and grow after the type of various forms of connective-tissue substances, or repeat one of the elementary tissues, while the imperfect histoid tumors are formed after the type of embryonal connective tissue.

*B.* Organoid tumors. *a.* Perfect: Adenoma, repeating the structure of a gland; papilloma, repeating the structure of the skin or mucous membrane. *b.* Imperfect: epithelioma, repeating imperfectly the structure of the skin or mucous membrane; carcinoma (glandular), repeating imperfectly the structure of a gland.

The perfect organoid tumors are formed, then, after the type of a typical epithelial structure, while the imperfect are a typical structure.

*C.* Paratoid tumors are represented only by dermoid cysts and made up of a number of separate tissues, as fat, cartilage, bone, teeth, or hair, enveloped in a capsule.

In the short time allotted to me I can hardly more than enumerate some of the more important tumors, giving a very brief outline of some of them.

Fibroma: Connective-tissue tumor, after the type of fibrous tissue. Common in domestic animals, particularly the dog and horse; may grow in any part of the body where connective tissue is found; usually single, but may be multiple; two varieties, hard and soft. Microscopy: Hard fibroma has many fibres running in various directions, few cells and bloodvessels; soft fibroma has many cells and fewer fibres, which are loosely arranged. The fibroma grows, generally, as a nodule, but occasionally may be pedunculated. Neoplasm: Fibromas may be found also in old scars, sometimes following castration or as keloid fibroma of the skin. The fibroma combines readily with the myoma, more seldom with myxoma, lipoma-sarcoma. The ordinary shoe-boil becomes eventually a fibromyoma. Degenerative processes are very common in fibromas: calcareous, mucoid fatty, and telangiectatic.

Myxoma: Connective-tissue tumors after the type of mucous tissue. Is not as common in animals as in man. A typical myxoma is soft and often hangs on a narrow pedicle, as a polyp from mucous membrane. They occur also in connection with the mammary gland, brain, and spinal cord. Microscopically they consist of stellate or spindle-shaped connective-tissue cells, which lie within a homogeneous, somewhat refractive or gelatinous matrix. Combines with fibroma and lipoma, but is also one of the invariable constituents of mixed tumors of the parotid gland or the testicles, and from the various tissues of the combination called adeno-fibro-myxochondro-carcinomatosis.

